# Hydrogels Formed by the Self‐Assembly of Collagen‐Mimetic Peptides With a Constrained Backbone Structure

**DOI:** 10.1002/chem.202503278

**Published:** 2026-01-07

**Authors:** Moeka Noto, Kazunori K. Fujii, Yuetsu Shu, Takashi Hiroi, Takaki Koide

**Affiliations:** ^1^ Department of Chemistry and Biochemistry School of Advanced Science and Engineering Waseda University Tokyo Japan

**Keywords:** collagen, peptide, self‐assembly, gel, triple helix

## Abstract

Self‐assembling peptides have attracted increasing attention as building blocks for developing functional materials. Among them, collagen‐mimetic peptides (CMPs) represent a class of self‐assembling systems. However, although short‐chain CMPs spontaneously fold into thermodynamically stable triple‐helical trimers, they fail to further undergo supramolecular assembly to form hydrogels. We report a new strategy for supramolecular hydrogel formation based on CMPs. The designed peptide, termed kinkCMP, features a kinked backbone introduced at the center of the sequence via adjacent disulfide bonds. The kinkCMP forms a gel, and its gel denaturation temperature can be tuned by varying the peptide chain length. Moreover, the hydrogel can be used as a cell culture scaffold. These results provide a versatile approach for constructing supramolecular hydrogels based on CMPs, expanding the design strategies for peptide‐based functional materials.

## Graphical abstract

Kinked collagen‐mimetic peptides (kinkCMPs), whose backbone is constrained by a disulfide bond, are designed to induce hydrogel formation. The kinked backbone promotes supramolecular assembly into tunable hydrogels with thermal stability depending on length. The resulting gels are also applicable as cell culture scaffolds, providing a new strategy for peptide‐based biomaterials.

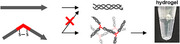

## 1 Introduction

Self‐assembling peptides are attractive building blocks for bottom‐up fabrication of functional materials, owing to their high design flexibility, and intrinsic ability to form higher‐order structures spontaneously. These peptides can form various secondary structures, including α‐helices and β‐strands driven by noncovalent supramolecular interactions such as electrostatic interactions, hydrogen bonding, hydrophilic and hydrophobic forces, van der Waals interactions, and π–π stacking. Consequently, they are capable of self‐assembling into a wide range of nano‐ and micro‐scale architectures [1, 2, 3]. Supramolecular peptide‐based materials have the potential to mimic the structural and dynamic features of native proteins and biological microenvironments. Therefore, when used as hydrogels, they offer promising biomedical applications such as carriers for sustained and controlled drug release [4], scaffolds for cell culture, and materials for tissue regeneration [5, 6].

Self‐assembling peptides based on collagen‐mimetic peptides (CMPs) have also attracted attention as components of biomaterials [7, 8, 9, 10]. Collagen, the major structural protein in the extracellular matrix, is composed of three polypeptide chains that intertwine to form a characteristic triple‐helical structure. Each chain contains repetitive (Xaa‐Yaa‐Gly) amino acid sequences, in which Xaa and Yaa are most commonly (2*S*)‐proline (Pro, P) and (2*S*,4*R*)‐4‐hydroxyproline (Hyp, O), respectively, followed by a glycine (Gly, G) residue that is essential for the tight packing of the triple helix. Each chain has a left‐handed polyproline II (PPII) conformation, and three such chains wind around one another in a staggered manner—offset by one residue—to form a right‐handed triple helix, defining the leading, middle, and trailing chains within the structure. CMPs, which reproduce this triple‐helical motif, typically consist of several tens of amino acid residues arranged in repetitive Xaa‐Yaa‐Gly triplets and are commonly synthesized through chemical methods. When CMPs are designed, the (Pro‐Hyp‐Gly) triplet—the most abundant one in native collagen—is frequently used because of its helix‐stabilizing effect. Historically, CMPs have been extensively used as models to elucidate the structural and thermodynamic properties of collagen triple helices [11, 12, 13, 14, 15, 16, 17, 18].

Short, chemically synthesizable CMPs tend to spontaneously form thermodynamically stable triple‐helical trimers. However, researchers are attempting to use their triple helix‐forming ability to construct higher‐order self‐assembled structures. One effective and simple strategy is connecting short triple helices to one another by introducing chemical linkages such as covalent bonds [19], cation–π interactions [20], and metal‐coordination [21]. Ion pair formations [22] between neighboring peptides are also useful in supramolecular formation. The other is the “sticky‐end assembly” system, similar to Woolfson's elongating α‐helical coiled‐coils [23], which enables staggered and hence elongated triple helix‐formation, yielding supramolecular structures [24, 25].

Some CMP‐based supramolecular designs have successfully provided gelation ability, which is a beneficial property in biomedical applications. Raines’ group and ours independently achieved hydrogel formation via sticky‐end assembly among disulfide‐bonded CMP [26] trimers with axial strand offsets [27, 28, 29, 30]. In this system, the strand offsets prevent perfectly aligned triple helix‐formation and force the peptides to form intermolecular triple helices with elongated chains. Hartgerink and co‐workers designed peptides capable of elongating the triple helix along the helical axis by introducing strand offsets *via* regular salt‐bridge formation rather than covalent bonds. These peptides also engage in lateral salt‐bridge interactions, promoting lateral self‐assembly. Consequently, a self‐supporting hydrogel with sufficient mechanical strength forms [31]. Raines and co‐workers refined this strategy and achieved gelation with a CMP as short as 33 residues by introducing symmetry to maximize the contribution of axial salt bridges to the thermostability of the assembly [32].

In this study, we report the design and preparation of a new supramolecular gel based on CMPs. As described above, short CMPs spontaneously fold into thermodynamically stable triple‐helical trimers. In contrast, gelatin, which is obtained by denaturing natural collagen, consists of long chains of over 1000 amino acid residues. When the gelatin refolds, these long chains cannot find their most stable structure, but undergo kinetically trapped intermolecular triple helix‐formation, resulting in supramolecular assembly and subsequent hydrogel formation. We hypothesized that CMPs incorporating a kinked backbone structure in the central region of the chain (kinkCMPs) would prevent the formation of trimeric triple helices, thereby enabling intermolecular folding similar to that of gelatin. Within the collagen triple helix, each polypeptide has a left‐handed PPII‐like helix, and all amide bonds assume the *trans* configuration [33]. In this study, we designed kinkCMPs in which the amide bond is fixed in the *cis* configuration to constrain the backbone conformation [34]. For kinkCMPs with a *cis*‐peptide bond, the formation of the otherwise most stable trimers was expected to be suppressed, favoring the formation of triple helices between adjacent molecules. Next, we describe the design, synthesis, hydrogel‐forming ability, and structural characteristics of kinkCMPs, and their applicability in cell culture systems.

## 2 Results and Discussion

### 2.1 Design of KinkCMPs

Peptides were designed to incorporate a kinked structure within the central region and possible triple helix‐forming regions in the flanking segments (Table 1, k6, k6R). Two adjacent cysteine (Cys) residues were introduced into the central region of the tandem Pro‐Hyp‐Gly repeats. The kinked structure was generated *via* a disulfide bond between these Cys residues, which constrained the amide bonds in the *cis* configuration. This region is therefore expected to be unable to form either a PPII‐like helix or, consequently, a triple helix. Peptide (l6R), in which the side‐chain thiol groups of the Cys residues were protected with acetamidomethyl (Acm) groups, was used as a control lacking a disulfide bond.

**TABLE 1:** List of peptides synthesized.

Peptide	Sequence
**k6**	$\mathrm{Ac}-{\left(\mathrm{POG}\right)}_{6}-\mathrm{CCG}-{\left(\mathrm{POG}\right)}_{5}-\mathrm{PO}-\mathrm{amide}$
**k6R**	$\mathrm{Ac}-{\left(\mathrm{POG}\right)}_{3}-\mathrm{PRG}-{\left(\mathrm{POG}\right)}_{2}-\mathrm{CCG}-{\left(\mathrm{POG}\right)}_{2}-\mathrm{PRG}-{\left(\mathrm{POG}\right)}_{2}-\mathrm{PO}-\mathrm{amide}$
**l6R**	$\mathrm{Ac}-{\left(\mathrm{POG}\right)}_{3}-\mathrm{PRG}-{\left(\mathrm{POG}\right)}_{2}-C(\mathrm{Acm})C(\mathrm{Acm})G-{\left(\mathrm{POG}\right)}_{2}-\mathrm{PRG}-{\left(\mathrm{POG}\right)}_{2}-\mathrm{PO}-\mathrm{amide}$

“C” indicates a half‐cystine residue.

### 2.2 Synthesis of KinkCMP

**SCHEME 1:**
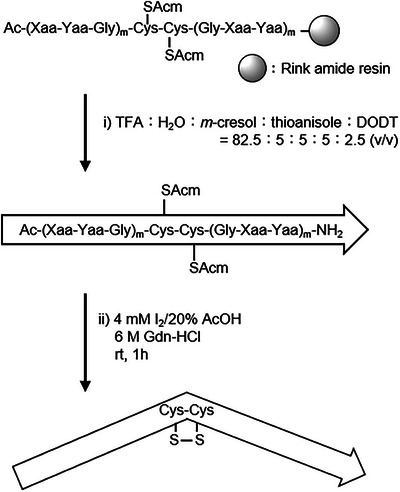
Synthetic scheme of kinkCMP.

**FIGURE 1:**
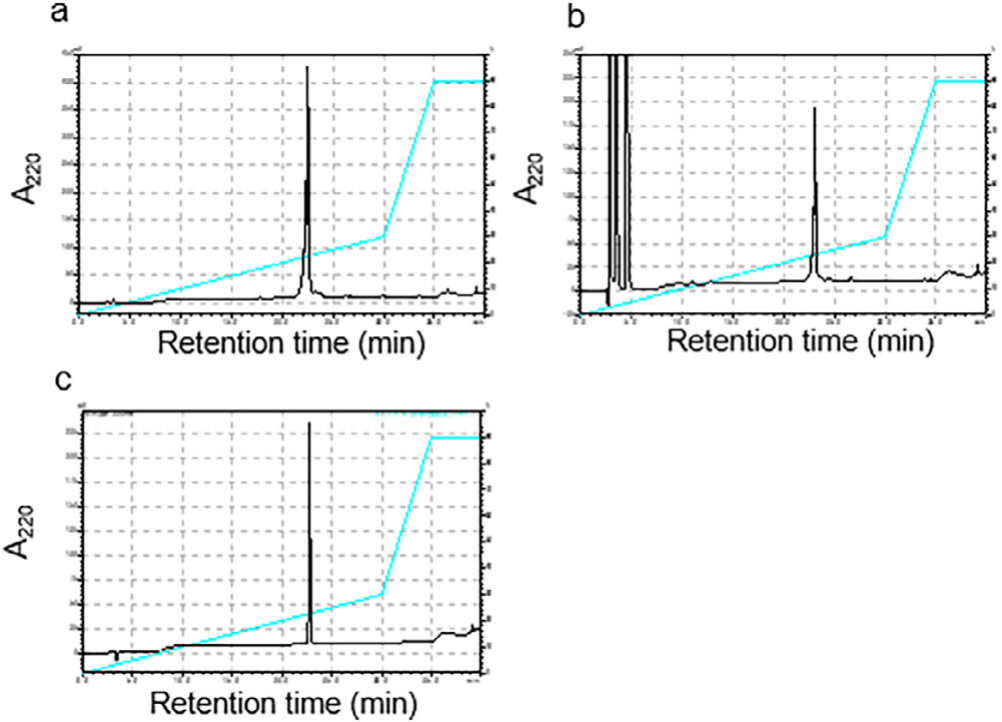
RP‐HPLC profile of each step in k6R synthesis. (a) Crude product of l6R after step i. (b) Crude product of k6R after step ii. (c) Purified k6R. Column: Cosmosil 5C_18_‐ARII (4.6 i.d. × 250 mm). Gradient: 0%−30% CH_3_CN in H_2_O (both containing 0.05% TFA) for 30 min at 60 °C. Flow rate: 1.0 *mL*/min. Absorbance: 220 nm.

Peptide chains were synthesized using the 9‐fluorenylmethoxycarbonyl (Fmoc) ‐based solid‐phase method. A blockwise condensation with Fmoc‐Pro‐Hyp‐Gly‐OH reduced the number of coupling steps and facilitated purification of the final products. The linear CMPs were obtained after treatment with a trifluoroacetic acid (TFA) based deprotection cocktail (Scheme 1, step i, Figure 1a). Intramolecular disulfide bond formation between adjacent Cys(Acm) residues was achieved by treating the peptide with excess iodine (I_2_) in a mixed solvent of acetic acid (AcOH) and water (Scheme 1, step ii, Figure 1b). In step ii, guanidinium hydrochloride (Gdn‐HCl) was added to the solvent to prevent possible triple helix‐formation. All peptides were purified using reversed‐phase high‐performance liquid chromatography (RP‐HPLC) to obtain a single peak and characterized *via* electrospray ionization time‐of‐flight mass spectrometry (ESI‐TOF MS) and matrix‐assisted laser desorption‐ionization time‐of‐flight mass spectrometry (MALDI‐TOF MS) (Figures 1c, S1, and S2).

### 2.3 Gel‐Forming Property of KinkCMPs

**FIGURE 2:**
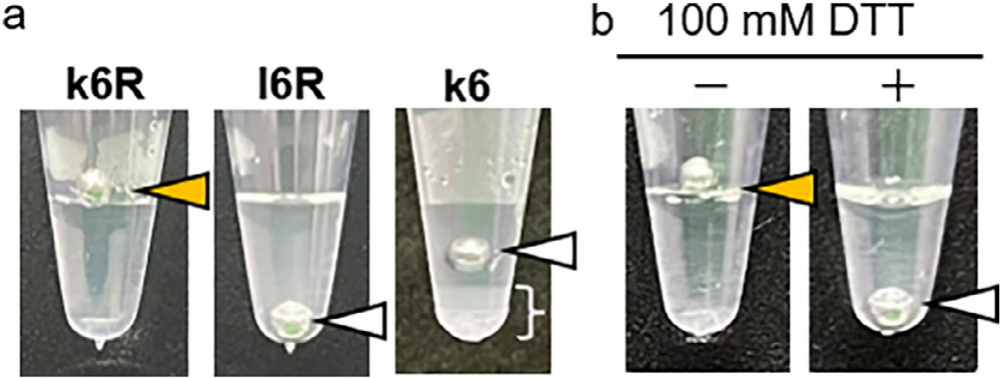
Hydrogel‐forming properties of the synthesized peptide. (a) Gel‐forming properties. All peptides (60 mg/*mL*) were heated at 95°C for 5 min and subsequently stored at 4°C. Gel formation and dissolution were evaluated by placing a stainless steel ball on the surface. White precipitate was observed for k6 in the region indicated by the white bracket. (b) Effect of DTT treatment on gel stability. k6R was dissolved in water at 60 mg/*mL* in the absence (‐) or presence (+) of 100 mM DTT and annealed under the same conditions, and its hydrogel formation was assessed. Arrowheads show the positions of the stainless‐steel balls: yellow arrowheads indicate hydrogel formation, and white ones indicate no hydrogel formation.

The hydrogel‐forming ability of the kinkCMPs was investigated. All peptides were dissolved in water at 60 mg/*mL* by heating, incubated at 95°C for 5 min, and then refolded at 4°C for 12 h. Gel formation was assessed in terms of the ability of a stainless steel ball [35] to remain on the surface. Under these conditions, **k6R** formed a hydrogel, whereas **l6R** did not (Figure 2a). When **k6R** was annealed in the presence of 100 mM dithiothreitol (DTT) to reduce disulfide bonds, no hydrogel formation was observed (Figure 2b). These results indicate the critical role of the kinked backbone introduced by a vicinal disulfide bond in promoting hydrogel formation. White precipitate formation with weaker gel formation was observed for **k6** (white bracket in Figure 2a), likely resulting from lateral aggregation of triple‐helical structures composed solely of repeating Pro‐Hyp‐Gly sequences, as reported previously [29, 36]. Thus, Arg‐containing peptides were used in subsequent experiments.

### 2.4 Characterization of Peptide Conformation and Analysis of Supramolecular Size

**FIGURE 3:**
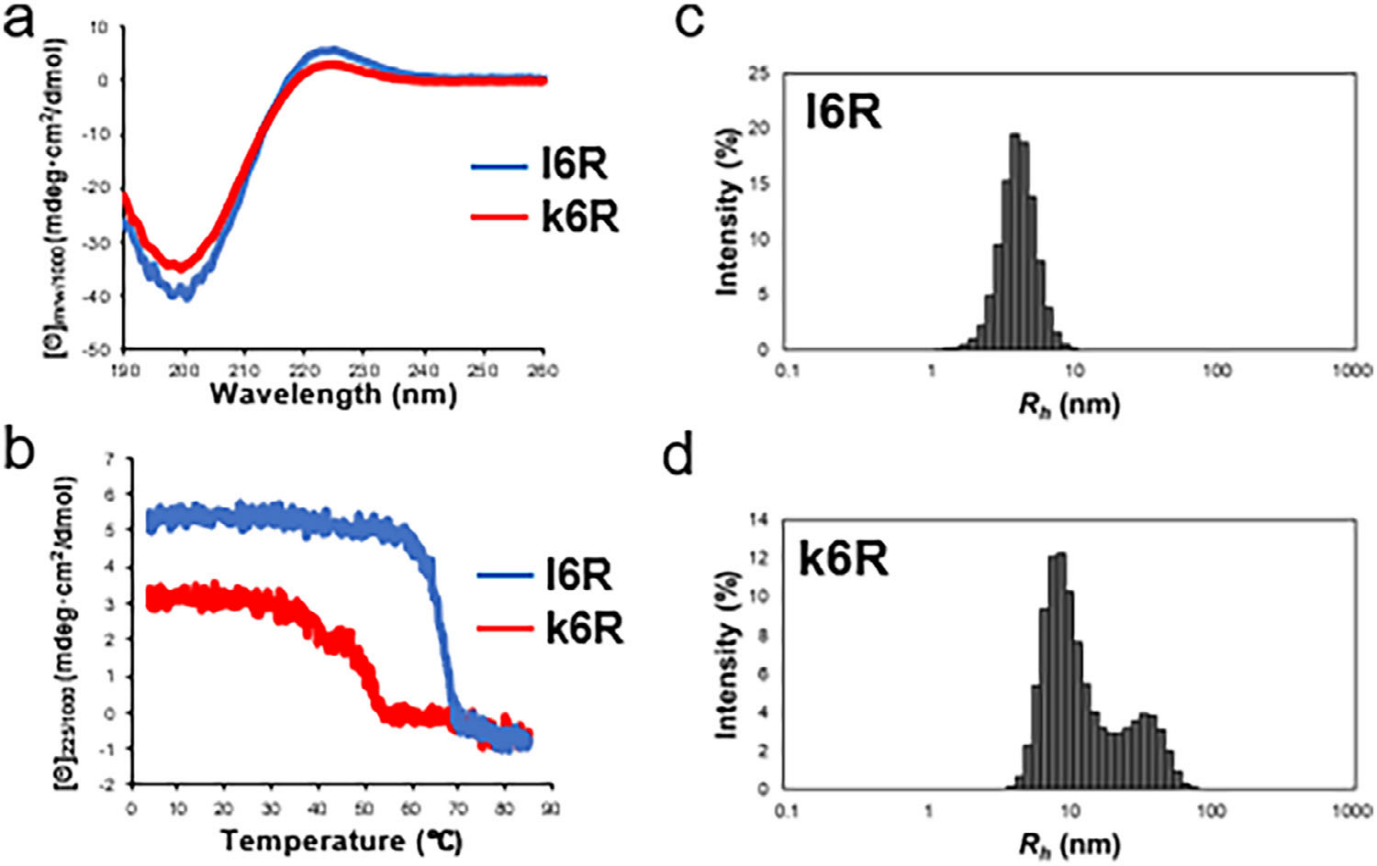
CD profiles and DLS analysis of the synthetic peptides. a) CD spectra recorded at 4°C. b) Thermal melting curves of the triple helices. The temperature was raised at a rate of 18°C/h. Peptide solutions were prepared by dissolving each peptide in water at a concentration of 0.5 mg/*mL*, followed by annealing under the same conditions as described above.

Conformational analysis was performed using circular dichroism (CD) spectroscopy to investigate whether hydrogel formation results from supramolecular assembly *via* intermolecular triple‐helical folding. Aqueous solutions of annealed **l6R** and **k6R** peptides (0.5 mg/*mL*) were used. At this concentration, no hydrogel formation was observed. Both **l6R** and **k6R** exhibited a positive signal at 224 nm and a negative signal at 200 nm around 4°C (Figure 3a), indicating the adoption of triple‐helical conformations. The *R_pn_* value, which is defined as the ratio of the positive to the negative peak intensity [37] and is a measure of triple helix content, was 0.13 and 0.092 for **l6R** and **k6R**, respectively. Conformational changes were further evaluated by monitoring [θ]_225_ as the temperature increased. [θ]_225_ values of **l6R** cooperatively decreased with increasing temperature, with a melting temperature (*T_m_*) of 67°C (Figure 3b). In contrast, the melting curve of **k6R** exhibited a more complex transition, consisting of at least two components, with the triple‐helical structure unfolding at a lower temperature than that of **l6R**. The *R_pn_* value of **l6R** is comparable to that of (Pro‐Hyp‐Gly)_10_ and native collagen (0.13). This, together with the single‐transition melting curve of **l6R**, suggests that **l6R** has a conventional trimeric triple‐helical structure [38]. In contrast, the more complex melting curve for **k6R** indicates the presence of multiple supramolecular species formed through intermolecular triple‐helical assembly. This behavior likely arises from the kinked backbone of **k6R**, which inhibits intramolecular trimer formation and instead promotes triple helix‐formation among many adjacent molecules, resulting in higher‐order structures. In these assemblies, the kinked region in the middle of the chain is unable to participate in triple helix‐formation. These observations are consistent with the lower triple helix content and decreased transition temperature of **k6R** relative to those of **l6R**.

The particle sizes of the supramolecular assemblies were further evaluated using dynamic light scattering (DLS). Measurements were performed on peptides dissolved in PBS at a concentration of 3 mg/*mL* after annealing. In the **l6R** solution, a single species with a hydrodynamic radius (*R_h_*) of 3.8 nm was observed (Figure 3c). This value corresponds well to the Stokes radius of a trimeric triple helix (3.6 nm, calculated as described in the Supporting Information). In contrast, the **k6R** solution exhibited at least two larger prominent populations, with *R_h_* values of approximately 9.2 nm and 33.7 nm, displaying a broad size distribution. This indicates that **k6R** forms intermolecular triple helices and undergoes supramolecular assembly (Figure 3d).

The relative abundance of particles of each size was determined through DLS. Solutions of c) l6R and d) k6R prepared in PBS (3 mg/*mL*) were heated at 95°C for 5 min, filtered through a 0.22 µm PVDF filter to remove insoluble materials, and stored at 4°C overnight to allow them to fold.

### 2.5 Thermal Stability of Hydrogels Composed of KinkCMPs With Different Chain Lengths

To investigate the relationship between peptide chain length and the thermal stability of the resulting hydrogels, a series of peptides based on **k6R** was synthesized with varying numbers of (Pro‐Hyp‐Gly) repeats (Table 2).

**TABLE 2:** List of synthetic peptides with varying chain lengths.

Peptide	Sequence
**k4R**	$\mathrm{Ac}-{\left(\mathrm{POG}\right)}_{2}-\mathrm{PRG}-\mathrm{POG}-\mathrm{CCG}-\mathrm{POG}-\mathrm{PRG}-\mathrm{POG}-\mathrm{PO}-\mathrm{amide}$
**k7R**	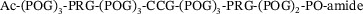
**k9R**	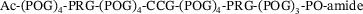

“C” indicates a half‐cystine residue.

**FIGURE 4:**
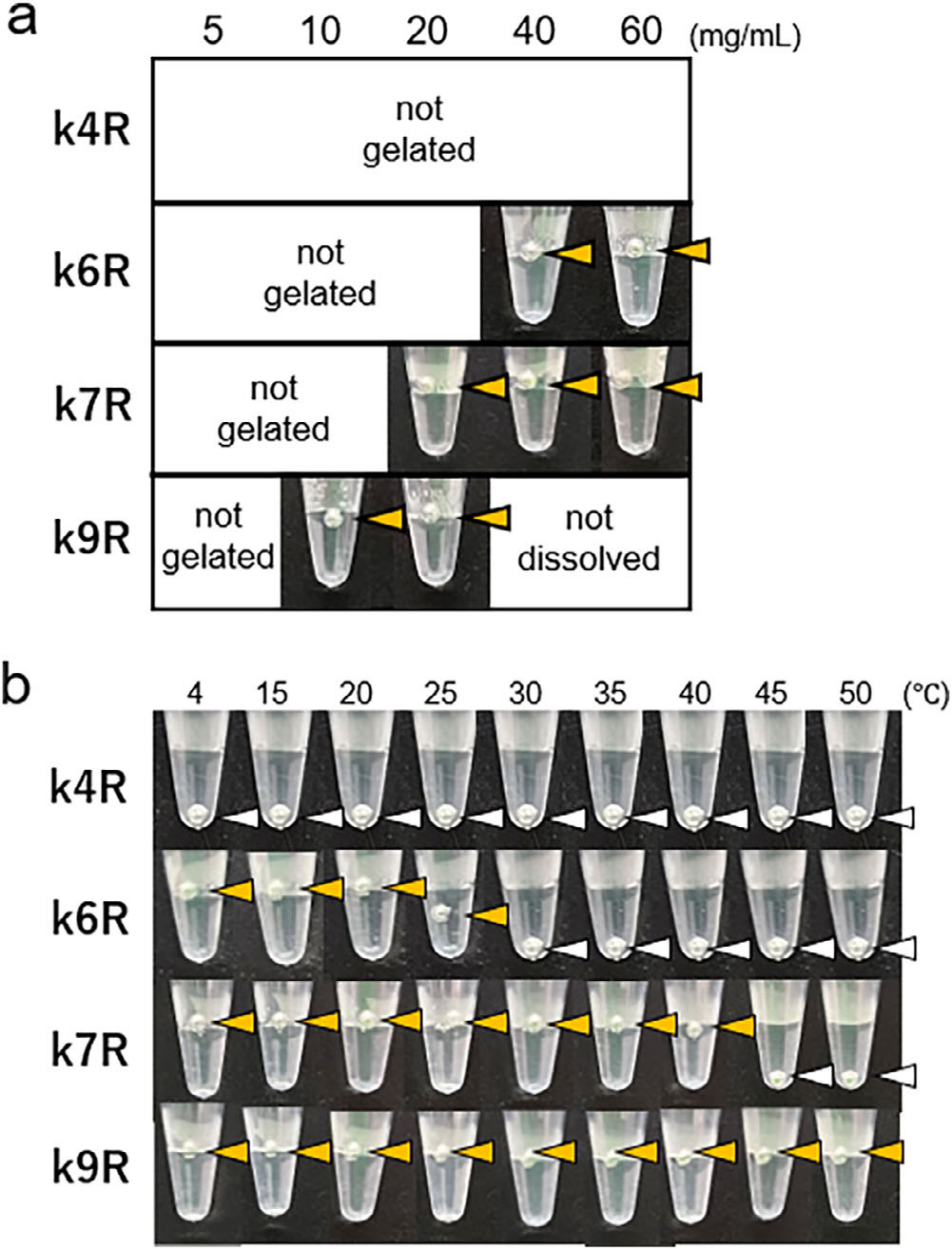
Relationship between peptide length and gelation properties. a) Determination of the minimum peptide concentration for gel formation. b) Effect of heating on gel stability. Solutions of k4R, k6R, and k7R were prepared in water at 40 mg/*mL*. k9R was dissolved at 20 mg/*mL*. Arrowheads show the positions of the stainless steel balls: yellow arrowheads indicate hydrogel formation, and white ones indicate no hydrogel formation.

These peptides, including **k6R**, were dissolved and annealed, and their hydrogel formation was evaluated. As shown in Figure 4a, **k4R** did not form a gel at the concentrations tested, whereas the minimum concentrations required for gel formation were determined to be 40, 20, and 10 mg/*mL* for **k6R**, **k7R**, and **k9R**, respectively. The thermal stability of the hydrogels was assessed by monitoring the sinking of stainless steel balls placed on the gel surfaces. *T_gel_* was defined as the temperature at which a stainless steel ball could no longer be supported at the gel surface. For hydrogels prepared at 40 mg/*mL* (except for **k9R**, which was only soluble at 20 mg/*mL*), *T_gel_* increased with peptide chain length. The *T_gel_* values of **k6R**, **k7R**, and **k9R** were 25 °C, 40 °C, and above 50 °C, respectively (Figure 4b).

CD spectra and temperature‐dependent monitoring of [θ]_225_ were performed for these peptides as described above (Figure S3). Both **k7R** and **k9R** exhibited negative and positive signals at 200 and 224 nm at 4°C, similar to those of **k6R**, indicating the existence of triple‐helical conformations. In contrast, **k4R** showed no positive peak around 225 nm, suggesting the absence of the triple helix. The melting curves of **k7R** and **k9R** also displayed complex transitions comprising at least two components, similar to that of **k6R**. Furthermore, DLS measurements showed broad distributions with major peaks at *R_h_* = 10.4 and 38.5 nm for **k7R** and *R_h_* = 8.0 and 41.9 nm for **k9R** (Figure S4), indicating supramolecular formation of these peptides similar to that of **k6R**.

### 2.6 Rheological Properties of KinkCMP Hydrogels

**FIGURE 5:**
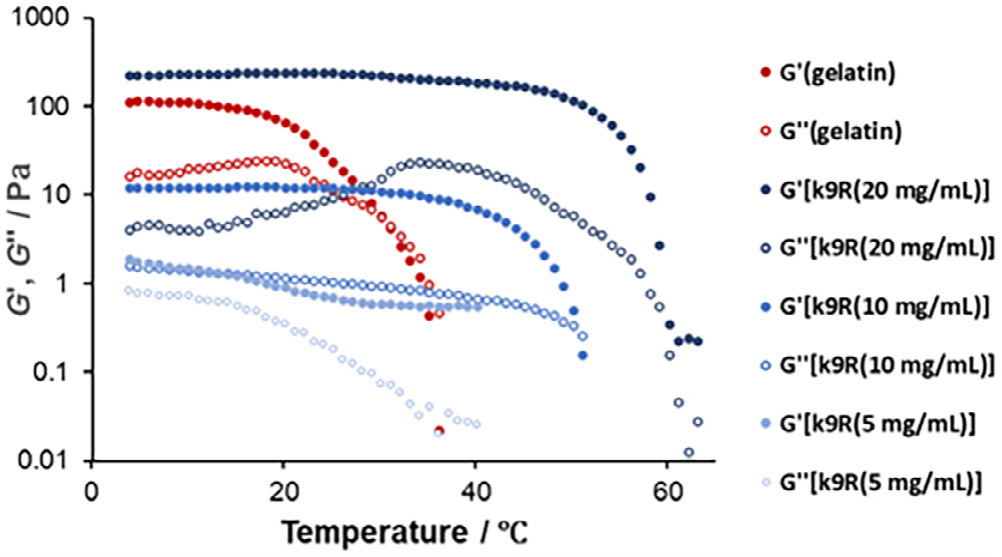
Temperature dependence of the storage (*G'*) and loss (*G''*) moduli of the k9R hydrogels and the gelatin gel. Measurements were performed using a 25‐mm parallel‐plate geometry with a 0.5–1.0 mm gap at 1 Hz and 1% strain, while ramping the temperature from 4°C.

The concentration and temperature dependences of the viscoelasticity of the kinkCMP hydrogels were quantitatively evaluated by rheological measurements. Figure 5 shows the temperature dependence of the storage modulus (*G'*) and loss modulus (*G''*) of **k9R** hydrogels with 20, 10, and 5 mg/*mL* concentrations. As a reference, the data for a 20 mg/*mL* gelatin gel is also shown. The *G'* values of the kinkCMP hydrogels increased nonlinearly with increasing concentration. In the case of 20 and 10 mg/*mL* samples, the crossovers of *G'* and *G''* were observed at 60°C and 50°C, respectively, which is the common criterion of sol‐gel transition [39]. The temperature dependence of the viscoelasticity of the kinkCMP hydrogels was similar to that of the gelatin gel, which shows the crossover of *G'* and *G''* at 30°C. These results are consistent with the thermal stability evaluated by the sinking behavior of the stainless steel ball (Figure 4b).

### 2.7 Scanning Electron Microscopy of the KinkCMP Hydrogel

**FIGURE 6:**
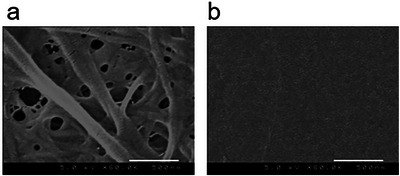
SEM images of the surface of (a) native collagen I and b) k9R hydrogel (scale bar  =  500 nm). Ten microliters of native collagen I (derived from bovine dermis) gel (2.5 mg/*mL*) and 10 µL of k9R peptide hydrogel (20 mg/*mL*) were prepared on a coverslip. The collagen gel was dehydrated using a graded series of ethanol/H_2_O solutions [50%, 60%, 70%, 80%, 90%, 100% (v/v)] and ethanol/*t*‐butanol solutions [50%, 60%, 70%, 80%, 90%, 100% (v/v)] followed by lyophilizing overnight. The k9R gel was also lyophilized overnight.

To examine the surface morphology, reconstituted collagen I gel and the kinkCMP hydrogel were observed *via* scanning electron microscopy (SEM). As shown in Figure 6, collagen fibrils with diameters in the range 50–100 nm were observed on the surface of the native collagen gel. In contrast, the **k9R** hydrogel did not exhibit nanoscale hierarchical structures but displayed only planar morphology with a fine, regular mesh‐like pattern. The result suggests that kinkCMP hydrogel formation is not driven by hierarchical self‐assembly, but rather by single‐level self‐assembly mediated through triple helix‐formation. Similar planar surface structures have also been found in our previous SEM observations of a hydrogel composed of a disulfide‐bonded triple‐helical CMP polymer [19].

### 2.8 Potential of KinkCMP Hydrogels as Cell Culture Substrates

**FIGURE 7:**
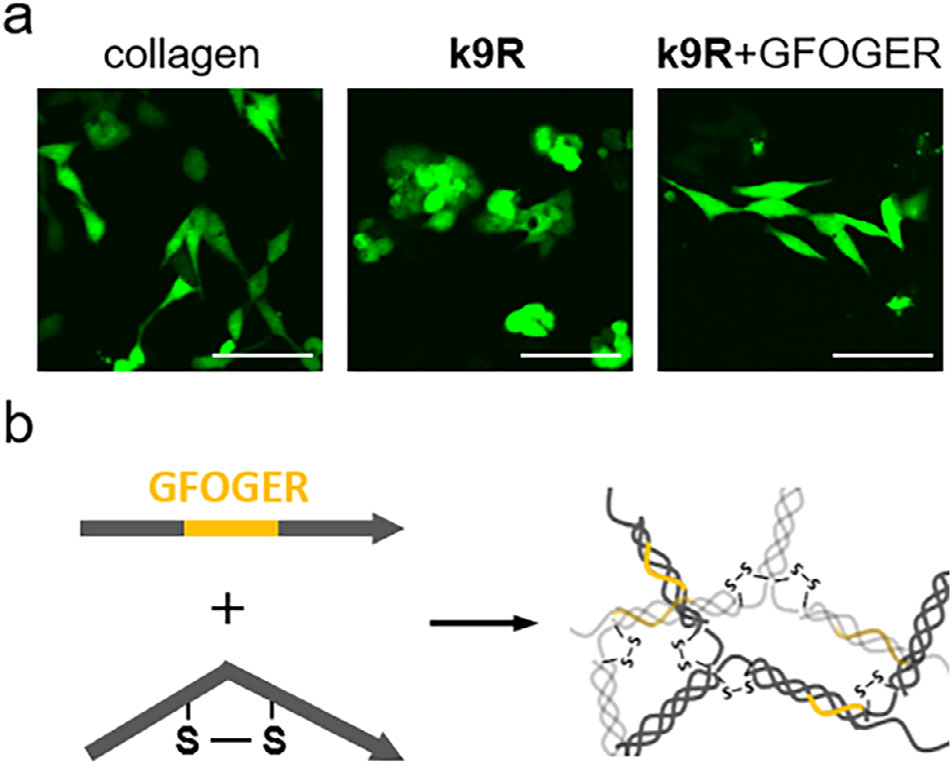
(a) Cell attachment to the kinkCMP hydrogels (scale bar  =  100 µm). GFP‐expressing HeLa cells were cultured on k9R gel (20 mg/*mL*), GFOGER‐containing k9R hydrogel (20 mg/*mL*), or native type I collagen gel (2.5 mg/*mL*q). Cells were cultured in D‐MEM supplemented with 10% (v/v) fetal bovine serum (FBS), 100 units/*mL* of penicillin, and 100 µg/*mL* of streptomycin. The cells were maintained at 37°C in a humidified 5% CO_2_/air atmosphere. (b) Illustration of a functionalized hydrogel with linear peptides containing the integrin‐binding motif (GFOGER).

The applicability of the synthetic kinkCMP hydrogels to cell culture systems was evaluated. We also attempted functionalization of the hydrogel by incorporating a functional triple‐helical peptide motif such as integrin‐binding sequences. Here we used a representative collagen‐binding integrin recognition sequence, Gly‐Phe‐Hyp‐Gly‐Glu‐Arg (GFOGER) [40], expecting cell‐attachment activity. A linear triple helix‐forming peptide named **Sol‐GFOGER** [Pro‐Hyp‐(Gly‐Pro‐Hyp)_5_‐Gly‐Phe‐Hyp‐Gly‐Glu‐Arg‐(Gly‐Pro‐Hyp)_5_‐Pro‐Tyr‐amide] was separately synthesized (Figure S1). To make the GFOGER‐containing **k9R** hydrogel, a mixture with a **k9R**‐to‐**Sol‐GFOGER** mass ratio of 30:1 was dissolved in water and heated, followed by triple helix‐formation at 4°C overnight. The GFOGER‐containing **k9R** hydrogel remained intact up to at least 50 °C and maintained its gel structure sufficiently at 37°C, the typical temperature for a cell culture (Figure S5). GFP‐expressing HeLa cells were seeded onto the gels and incubated for 3 days at 37°C, after which adherent cells were observed through confocal fluorescence microscopy (Figure 7a). Overall, the cells adhered to the hydrogel surfaces and no harmful effects of the gel were visually observed, indicating that kinkCMP hydrogels can serve as scaffolds for cell culture. Moreover, the GFOGER‐containing **k9R** hydrogel exhibited markedly enhanced cell spreading compared with the **k9R** hydrogel.

These observations suggest that the enhanced cell adhesion was due to integrin recognition of triple‐helical GFOGER motifs present on the hydrogel surface. This implies that the linear GFOGER‐containing peptide, **Sol‐GFOGER**, co‐assembled with **k9R** into triple helices, becoming integrated into the supramolecular network (Figure 7b). This simple strategy for gel functionalization could also enable the formation of peptide hydrogels with diverse functionalities by incorporating other functional sequences found within the triple‐helical region of collagen, such as Arg‐Gly‐Gln‐Hyp‐Gly‐Val‐Met‐Gly‐Phe‐Hyp for von Willebrand factor [41], Gly‐Val‐Met‐Gly‐Phe‐Hyp for discoidin domain receptors [42], secreted protein that is acidic and rich in cysteine (SPARC) [43, 44], and Lys‐Gly‐His‐Arg‐Gly‐Phe for heparan sulfate proteoglycan and pigment epithelium‐derived factor [45, 46]. Under the standard cell culture condition, the **k9R** hydrogel gradually eroded and decreased in size over the course of several days. While the **k9R** hydrogel may be suitable as a short‐term carrier for cell transplantation, additional stabilization strategies would be required for long‐term culture applications.

## 3 Conclusion

In this study, we fabricated hydrogels based on synthetically accessible CMPs. The design concept of the peptides is straightforward: a kink structure, incapable of forming a triple helix, was introduced into the central region of the peptide chain. This structural constraint prevents the formation of perfectly aligned trimeric triple helices that would otherwise be thermodynamically favored. Consequently, the collagen‐like Xaa‐Yaa‐Gly sequences located at both the N‐ and C‐termini are driven to form intermolecular triple helices. Such kinetically trapped supramolecular assemblies give rise to hydrogels through a mechanism similar to that for gelatin gels derived from native collagen. Recent approaches to CMP‐based hydrogel design have focused on mimicking the higher‐order fibrillar organization of native collagen by introducing precisely designed interchain ionic pairs [31, 32]. In contrast, this study provides an alternative collagen‐inspired hydrogel system that accommodates a broader variety of collagen‐like amino acid sequences and whose gel denaturation temperature can be tuned simply by varying the peptide chain length.

## Conflicts of Interest

N. M., K. K. F., Y. S. and T. K. are named as inventors of kinkCMPs on a pending patent application jointly filed by Waseda University and Kola‐Gen Pharma Inc.

## Supporting information



The authors have cited additional references within the Supporting Information.
