# Supplementary material for: Hydrogels Formed by the Self‐Assembly of Collagen‐Mimetic Peptides With a Constrained Backbone Structure
Source: Chemistry. 2026 Jan 7;32(10):e03278. doi: 10.1002/chem.202503278 (PMC12995845; doi:10.1002/chem.202503278)
Supplement: Supplementary file 1 — The authors have cited additional references within the Supporting Information. [file CHEM-32-e03278-s001.docx]

**Chemistry－A European Journal**

Supporting Information

**Hydrogels Formed by the Self-Assembly of Collagen-Mimetic Peptides with a Constrained Backbone Structure**

Moeka Noto, Kazunori K. Fujii, Yuetsu Shu, Takashi Hiroi, Takaki Koide

Department of Chemistry and Biochemistry, School of Advanced Science and Engineering,

Waseda University, Shinjuku-ku, Tokyo 169-8555, Japan

**Table of contents**

1. List of names and abbreviations S2
2. Materials and methods
   1. Peptide synthesis and purification S4
   2. Preparation and evaluation of peptide hydrogels S4
   3. Circular dichroism (CD) spectroscopy S5
   4. Dynamic light scattering (DLS) S5
   5. Rheological measurement S7
   6. Scanning electron microscopy (SEM) S7
   7. Cell culture S7
   8. Cell adhesion assay S8
3. Supplementary analytical data
   1. Reversed-phase high-performance liquid chromatography (RP-HPLC) of the synthesized peptide S9
   2. MS charts of the synthetic peptides S10
   3. CD profiles of the synthetic peptides S14
   4. DLS analysis of the peptides S15
   5. Thermal stability of GFOGER-containing **k9R** hydrogel S16
   6. Stability of **k9R** hydrogel under the standard cell culture condition S16
4. References S17
5. **List of names and abbreviations**

Ac acetyl

Acm acetamidomethyl

Arg (R) arginine

CD circular dichroism

Cys (C) cysteine

DIC *N,N*′-diisopropylcarbodiimide

DLS dynamic light scattering

D-MEM Dulbecco’s modified Eagle’s medium

DMF *N,N*-dimethylformamide

DODT 3,6-dioxa-1,8-octanedithiol

EDTA ethylenediaminetetraacetic acid

ESI-TOF MS electrospray ionization time-of-flight mass spectrometry

FBS fetal bovine serum

Fmoc 9-fluorenylmethyloxycarbonyl

Gdn-HCl guanidinium hydrochloride

GFP green fluorescent protein

Glu (E) glutamic acid

Gly (G) glycine

HOBt 1-hydroxybenzotriazole

Hyp (O) (2S,4R)-4-hydroxyproline

MALDI-TOF MS matrix-assisted laser desorption-ionization time-of-flight mass spectrometry

Pbf 2,2,4,6,7-pentamethlydihydrobenzofuran-5-sulfonyl

PBS phosphate-buffered saline

PPII polyproline II

Pro (P) (2S)-proline

RP-HPLC reversed-phase high-performance liquid chromatography

SEM scanning electron microscope

tBu tert-butyl

TFA trifluoroacetic acid

1. **Materials and methods**
   1. **Peptide synthesis and purification**

Peptide chains were manually constructed according to the standard Fmoc-based solid-phase peptide synthesis protocol on Rink Amide AM resin LL (Novabiochem, San Diego, CA, USA). Coupling reactions were performed at room temperature for 2 h using 3–5 equivalents of Fmoc-amino acids or Fmoc-tripeptide in the presence of HOBt (Nacalai Tesque, Kyoto, Japan) and DIC (Fujifilm Wako Pure Chemical Industries, Osaka, Japan) in DMF. The following Fmoc-protected building blocks were used: Fmoc-Pro-OH, Fmoc-Hyp(*t*Bu)-OH, Fmoc-Gly-OH, Fmoc-Arg(Pbf)-OH, Fmoc-Cys(Acm)-OH, Fmoc-Glu-OH, and Fmoc-Pro-Hyp-Gly-OH.^[1]^ The peptides were deprotected and cleaved off from the resin with a cocktail of TFA (Nacalai Tesque, Kyoto, Japan)/*m*-cresol/thioanisole/H_2_O/DODT (82.5:5:5:5:2.5 v/v) at room temperature for 4 h.

To prepare kinkCMPs, disulfide bonds between adjacent cysteine residues were formed under the following conditions. The peptides were treated with 4 mM I_2_ in 20% (v/v) AcOH/H_2_O with 6 M Gdn-HCl at room temperature for 1 h. I_2_ was quenched with excess ascorbic acid. After being desalted with Sephadex G-25 (GE Healthcare Japan, Tokyo, Japan) using 0.05% (v/v) TFA/H_2_O as a solvent, the peptides were purified *via* RP-HPLC on a COSMOSIL 5C18-AR-II column (20 mm i.d. × 250 mm; Nacalai Tesque, Kyoto, Japan) at 60 °C with CH_3_CN (Merck, Darmstadt, Germany) in water, both containing 0.05% (v/v) TFA.

Mass spectrometry was performed with ESI-TOF MS [Compact (ESI), Bruker, Billerica, MA, USA] or MALDI-TOF MS (Autoflex MAX, Bruker) (Figure S2).

- 1. **Preparation and evaluation of peptide hydrogels**

PCR tubes containing aqueous solutions (30 µL) of peptides at various concentrations were heated at 95 °C for 5 min and subsequently stored at 4 °C. Gel formation and dissolution were evaluated by placing a stainless-steel ball (SUS440C, diameter = 1.5 mm; Funabe Seiko, Hyogo, Japan) on the gel surface. The thermal stability of the gels was evaluated using the same method after incubating the gels at specific temperatures for 5 min in a thermostated incubator. The temperature at which a stainless steel ball could no longer be supported at the gel surface was defined as *T_gel_*.

- 1. **Circular dichroism (CD) spectroscopy**

CD spectra were recorded on a J-820 CD spectropolarimeter (JASCO, Tokyo, Japan) equipped with a Peltier thermal controller, using a 0.5-mm quartz cuvette. The instrument was connected to a data station for signal averaging. Spectral intensity was converted into the mean residue weight ellipticity ([θ]_MRW_) of the collagenous (Gly-Xaa-Yaa)_n_ portion of the peptides. Peptides were dissolved in water (0.5 mg/mL) and heated at 95 °C for 5 min, followed by storage at 4 °C to allow them to fold. Data were obtained using continuous wavelength scans from 190 to 260 nm (Figure S3a). Thermal denaturation of the triple helix and PPII helix was monitored by following the [θ]_MRW_ value at 225 nm ([θ]_MRW,225_) while increasing the temperature from 4 to 85 °C at 18 °C per hour (Figure S3b).

- 1. **Dynamic light scattering (DLS)**

DLS analysis was carried out on a DLS Particle Size Analyzer (LB-550; Horiba, Ltd., Kyoto, Japan). Peptides were dissolved in PBS at concentrations of either 3.0 mg/mL (**k4R, k6R, k7R, and l6R**) or 1.0 mg/mL (**k9R**). All samples were heated at 95 °C for 5 min, filtered through a 0.22 µm PVDF filter to remove insoluble materials, and stored at 4 °C overnight to allow them to fold. The measurements were performed with 60 accumulations for each sample, and five determinations were recorded for each sample.

The hydrodynamic (Stokes) radius of the triple-helical peptide was calculated as follows:

[Length of the polyproline II (PPII) helix]

The contour length of a PPII helix was estimated from the number of residues and the rise per residue. For a peptide of *N* residues, the length *L* is given by:

$$\begin{aligned} L=N \times h \#\left( S1 \right) \end{aligned}$$

where $h=3.1 Å$ is the rise per residue for a PPII helix.^[2]^ For the **k9R** peptide consisting of 38 residues, the length is estimated to be:

$$\begin{aligned} L=38 \times3.1 Å \approx117.8 Å \left( \approx11.8 \mathrm{nm} \right) \#\left( S2 \right) \end{aligned}$$

[Hydrodynamic radius of the peptide]

The hydrodynamic (Stokes) radius was estimated from the translational diffusion coefficient using the Stokes–Einstein equation with Perrin’s correction factor for a prolate ellipsoid:

$$\begin{aligned} R_{S}=\frac{kT}{6\pi\eta D_{t}}P\left( \frac{a}{b} \right) \#\left( S3 \right) \end{aligned}$$

where *k* is the Boltzmann constant, *T* is the absolute temperature, *η* is the solvent viscosity, and *D_t_​* is the translational diffusion coefficient. The term *P(a/b)* represents the Perrin shape correction factor accounting for the deviation of a molecule from a perfect sphere, where *a*/*b* denotes the axial ratio of a prolate ellipsoid.

This study assumed a prolate ellipsoid with a long axis $a=6.0 \mathrm{nm}$ and a short axis $b=1.0 \mathrm{nm}$.
The corresponding Perrin factor P(6) was calculated according to the theoretical treatment described by *García de la Torre* and *Hernández-Cifre*^[3]^, which updates the formulation of hydrodynamic parameters for ellipsoidal particles based on the classical Perrin equation.

- 1. **Rheological measurement**

Rheological measurements were conducted using an MCR302e rheometer (Anton Paar, Austria) equipped with a 25 mm parallel plate. The composites of **k9R** with different concentrations (20, 10, and 5 mg/mL) were set on the aluminum stage at 4 °C. Gelatin (Fujifilm Wako Pure Chemical Industries, Osaka, Japan) solution was put on the stage at 37 °C, and the sample temperature was decreased to 4 °C. After the stabilization of the sample temperature by waiting for 5 minutes at 4 °C, the storage modulus (*G'*) and loss modulus (*G''*) were measured as a function of temperature. The sample temperature was increased at a rate of 2°C/minute, and the measurements were performed every 30 seconds. The oscillation frequency and the strain were set at 1 Hz and 1%, respectively. The sample thickness ranged from 0.5 to 1.0 mm, depending on the sample amount. The data points measured with enough torque (typically more than 1×10^−5^ mN·m) are shown in Figure 5.

- 1. **Scanning electron microscopy (SEM)**

Ten µL of acid-soluble collagen I derived from bovine dermis (Koken Co., Ltd., Tokyo, Japan) gel (2.5 mg/mL) and a **k9R** peptide hydrogel (20 mg/mL) were prepared on a coverslip. The collagen gel was prepared by treating native type I collagen in 20 mM phosphate buffer (pH 7.4) containing 100 mM NaCl at 37 °C for 30 min and was dehydrated using a graded series of H_2_O/ethanol solutions [50%, 60%, 70%, 80%, 90%, 100% (v/v)] and ethanol/*t*-butanol solutions [50%, 60%, 70%, 80%, 90%, 100% (v/v)] followed by lyophilizing overnight. The **k9R** gel was also lyophilized overnight. All samples were coated with platinum and observed under an SEM (S-3000N; Hitachi, Tokyo, Japan).

- 1. **Cell culture**

Human cervical adenocarcinoma HeLa cells constitutively expressing GFP were provided by Prof. Yasuhiko Terada (Waseda University). Cells were cultured in D-MEM (Fujifilm Wako Pure Chemical Industries, Osaka, Japan) supplemented with 10% (v/v) FBS (Thermo Fisher Scientific, Waltham, MA, USA), 100 units/mL penicillin, and 100 µg/mL streptomycin (Sigma-Aldrich, St. Louis, MO, USA). The cells were maintained at 37 °C in a humidified 5% CO_2_/air atmosphere.

- 1. **Cell adhesion assay**

Native type I collagen gels (2.5 mg/mL) and kinkCMP gels (20 mg/mL) with a diameter of 6 mm and thickness of 1 mm were prepared on a 35 mm glass-base dish (Iwaki, Tokyo, Japan). GFP-expressing HeLa cells were detached with 0.05% (w/v) trypsin/0.53 mM EDTA (Fujifilm Wako Pure Chemical Industries), collected *via* centrifugation (120 × *g*, 4 min at 24 °C), and resuspended in the assay medium [D-MEM containing 1% (v/v) FBS] to a concentration of 1 × 10^5^ cells/well. One mL of the suspended cells was seeded onto the gels and incubated at 37 °C in a humidified 5% CO_2_/air atmosphere for 3 days. Fluorescence images of cells on the gels were observed under a confocal fluorescence microscope (FLUOVIEW FV1000; Olympus, Tokyo, Japan).

1. **Supplementary analytical data**
   1. **Reversed-phase high-performance liquid chromatography (RP-HPLC) of the synthesized peptide**

**k6 k6R**

**
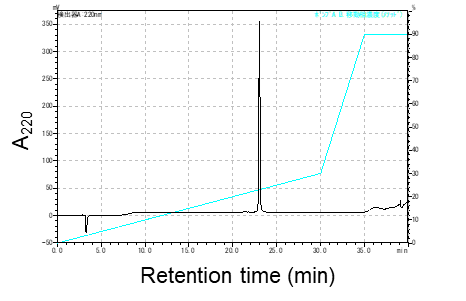

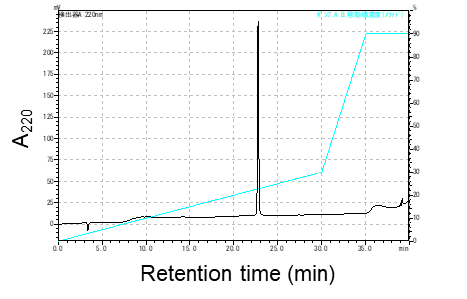
**

**l6R k4R**

**
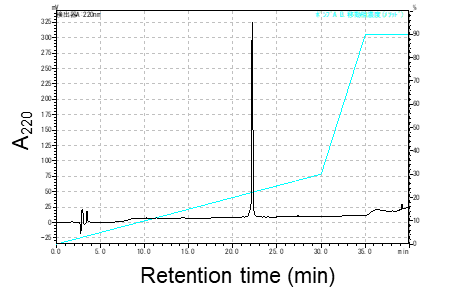
**
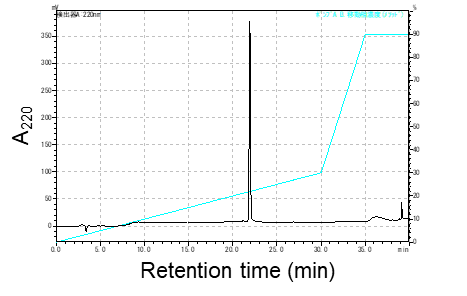


**k7R k9R**

**
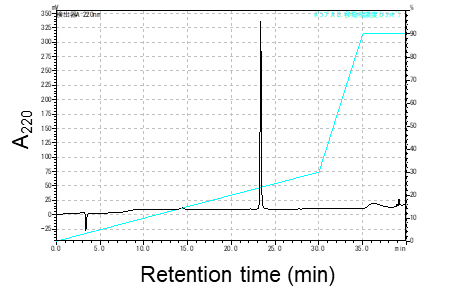

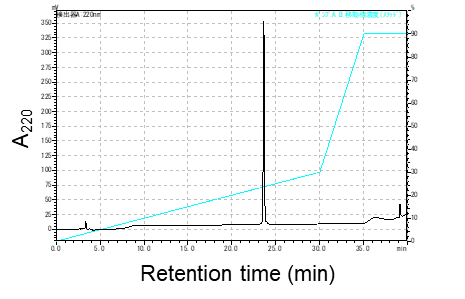
**

**Sol-GFOGER**

**
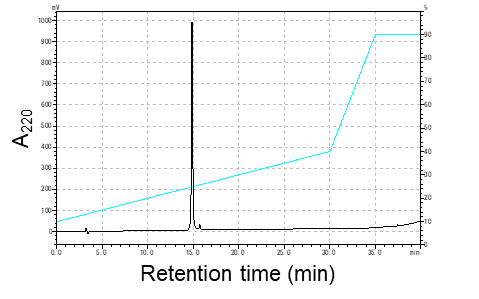
**

**Figure S1.** RP-HPLC profiles of the synthesized peptide

Column: Cosmosil 5C18-ARII (4.6 i.d. × 250 mm).

Gradient: 0%−30% or 10%−40% CH_3_CN in H_2_O (0.05% TFA) for 30 min at 60 °C. Flow rate: 1.0 mL/min. Absorbance: 220 nm.

- 1. **MS charts of the synthetic peptides**


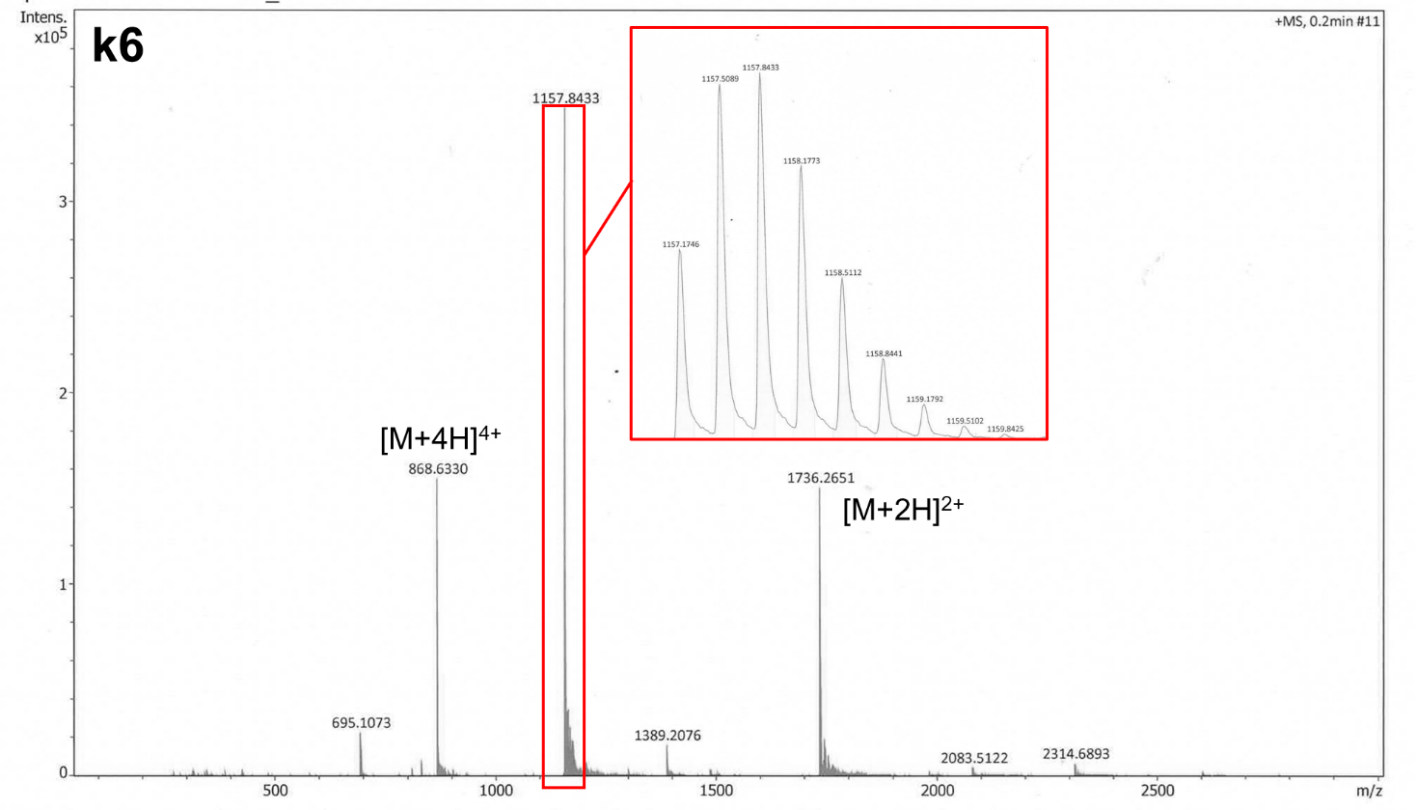


ESI-TOFMS (positive mode) m/z: [M+3H]^3+^ calculated for C_152_H_217_N_39_O_51_S_2_ = 1157.174, observed m/z = 1157.1746


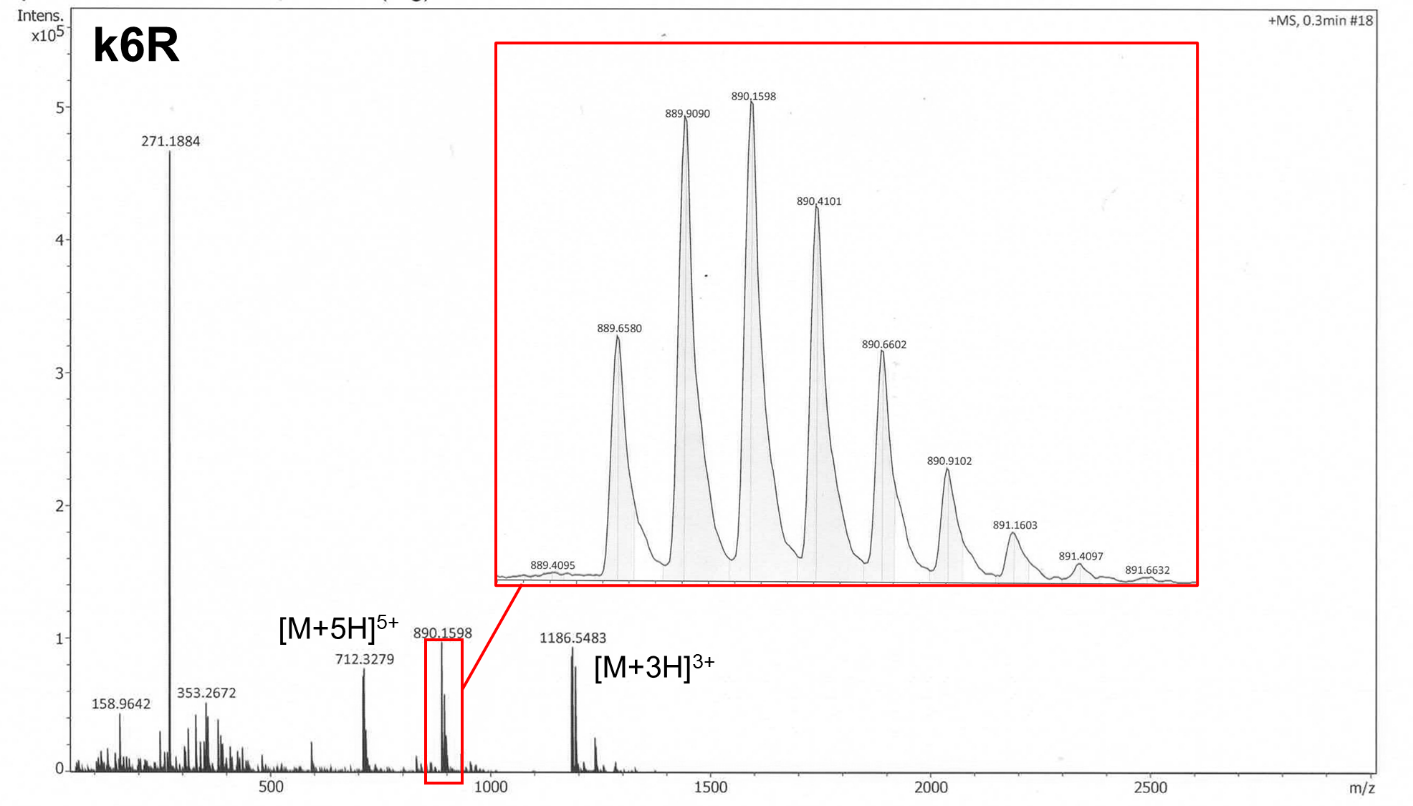


ESI-TOFMS (positive mode) m/z: [M+4H]^4+^ calculated for C_154_H_227_N_45_O_49_S_2_ = 889.659, observed m/z = 889.6580

**Figure S2.** MS charts of the synthetic peptides

**
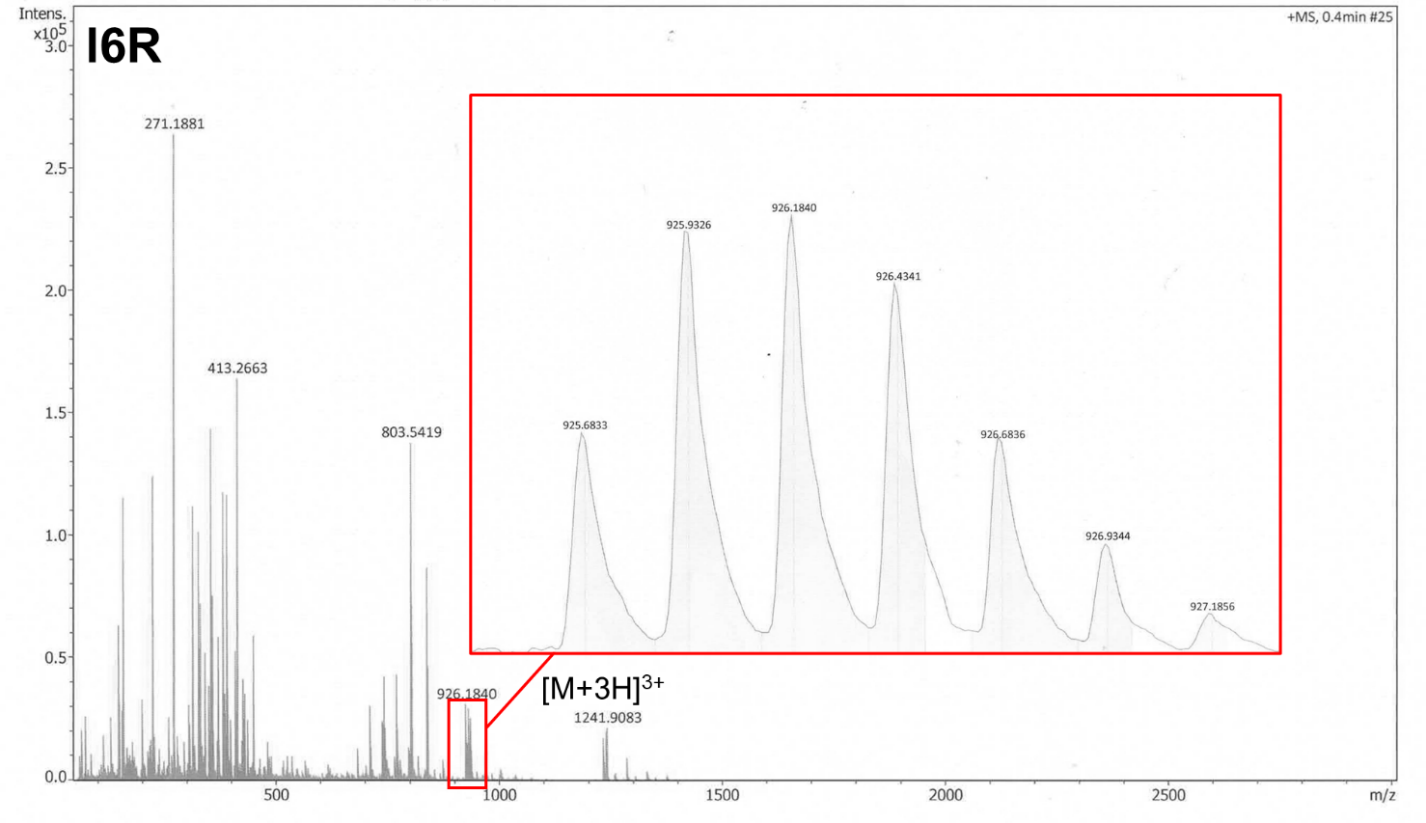
**

ESI-TOFMS (positive mode) m/z: [M+4H]^4+^ calculated for C_160_H_239_N_47_O_51_S_2_ = 925.681, observed m/z = 925.6833


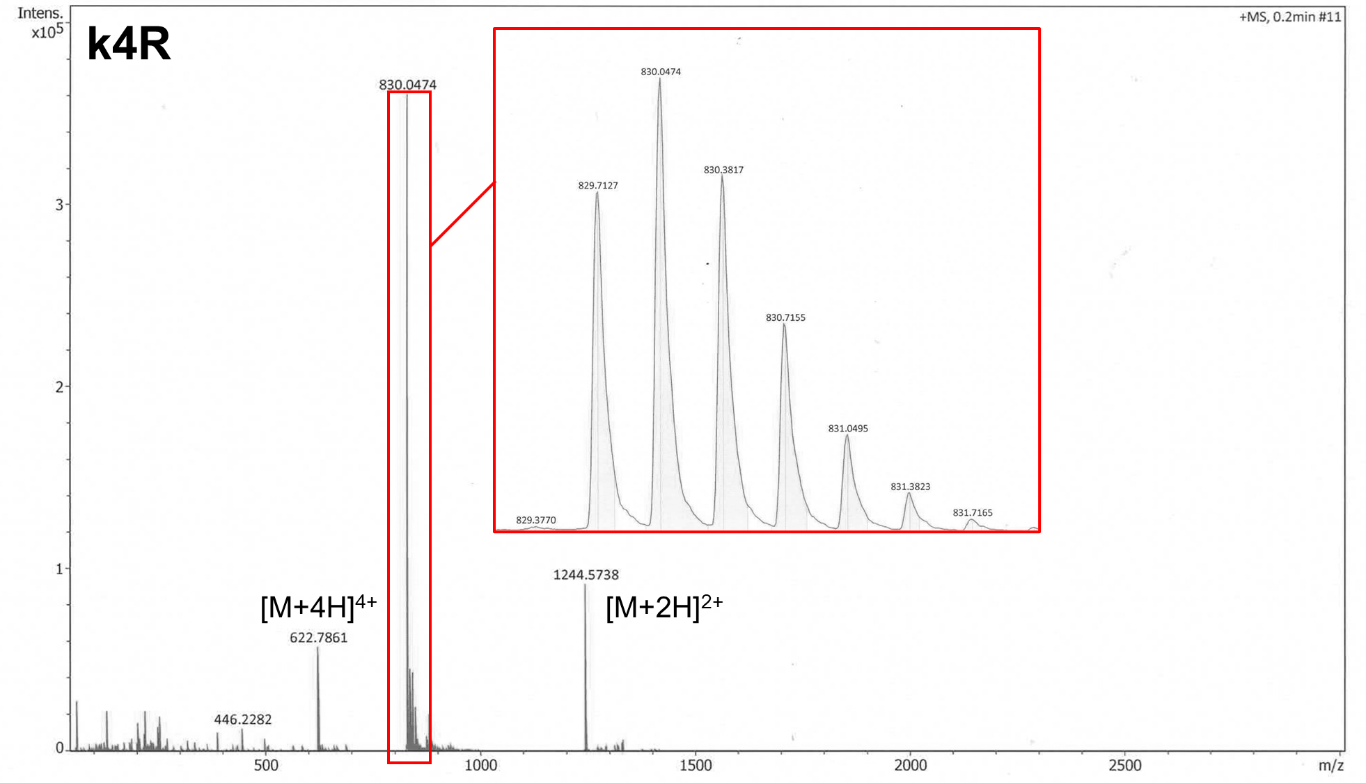


ESI-TOFMS (positive mode) m/z: [M+3H]^3+^ calculated for C_106_H_159_N_33_O_33_S_2_ = 829.714, observed m/z = 829.7127

**Figure S2.** (Continued)

**
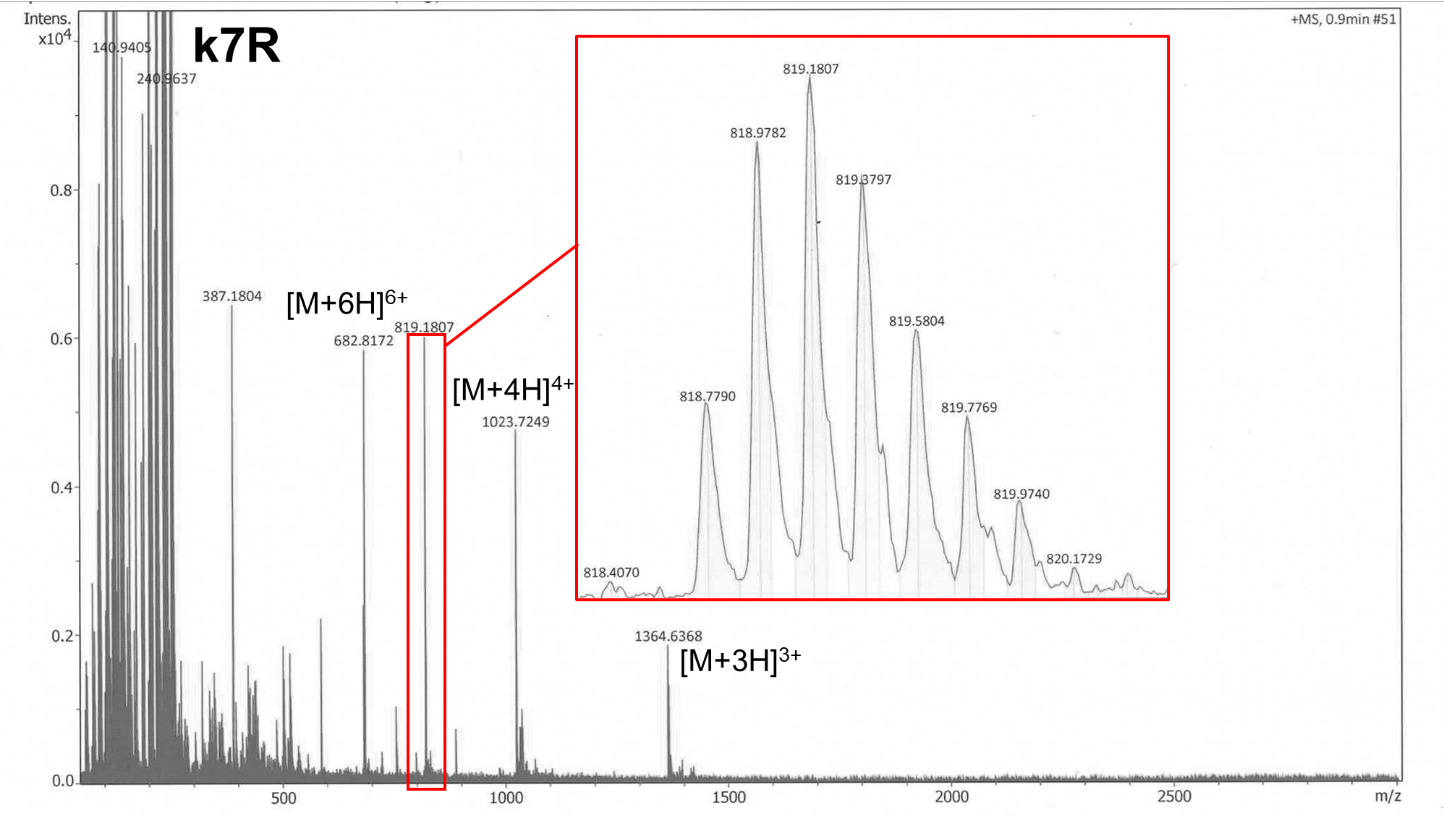
**

ESI-TOFMS (positive mode) m/z: [M+5H]^5+^ calculated for C_178_H_261_N_51_O_57_S_2_ = 818.777, observed m/z = 818.7790

**
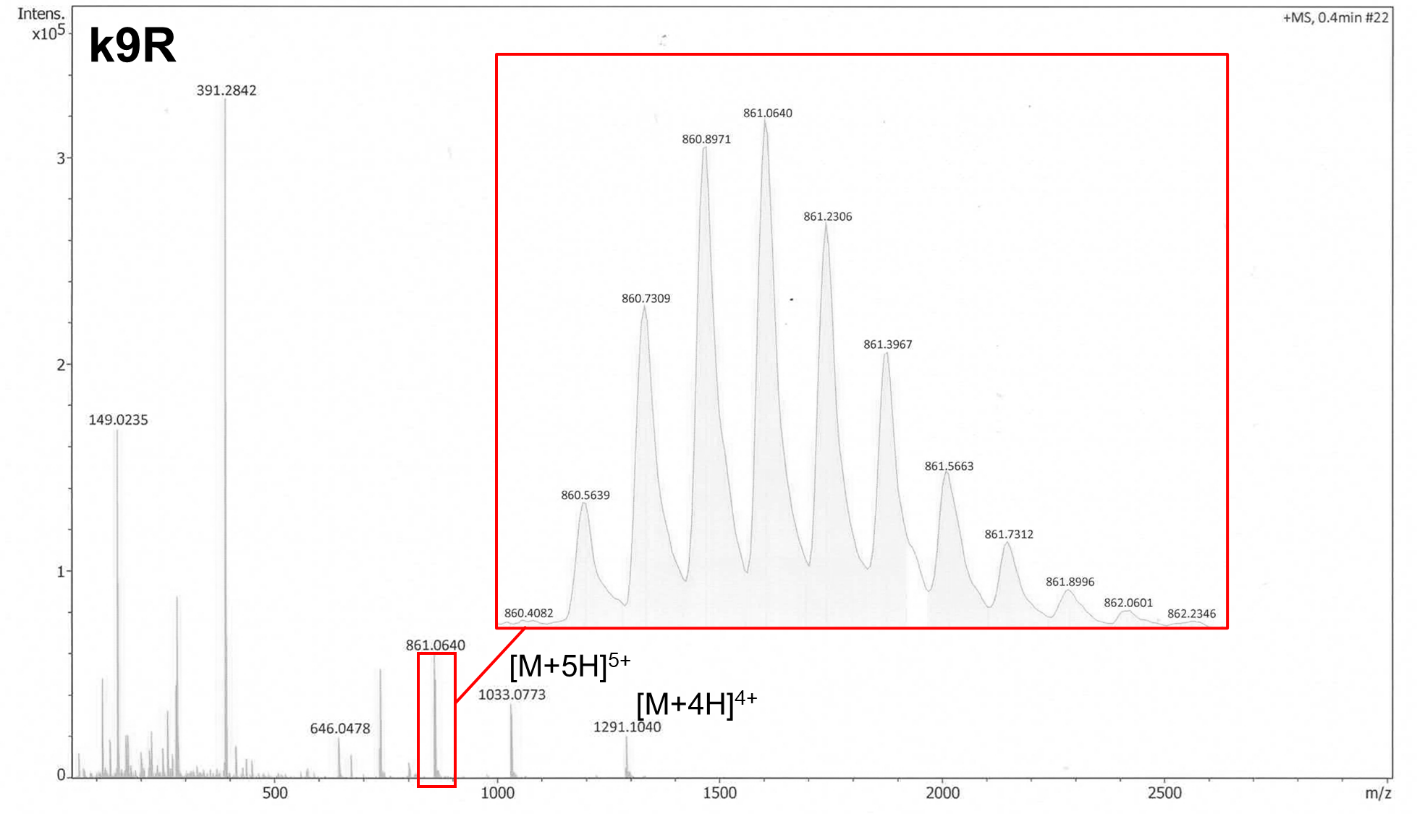
**

ESI-TOFMS (positive mode) m/z: [M+6H]^6+^ calculated for C_226_H_329_N_63_O_73_S_2_ = 860.563, observed m/z = 860.5639

**Figure S2.** (Continued)


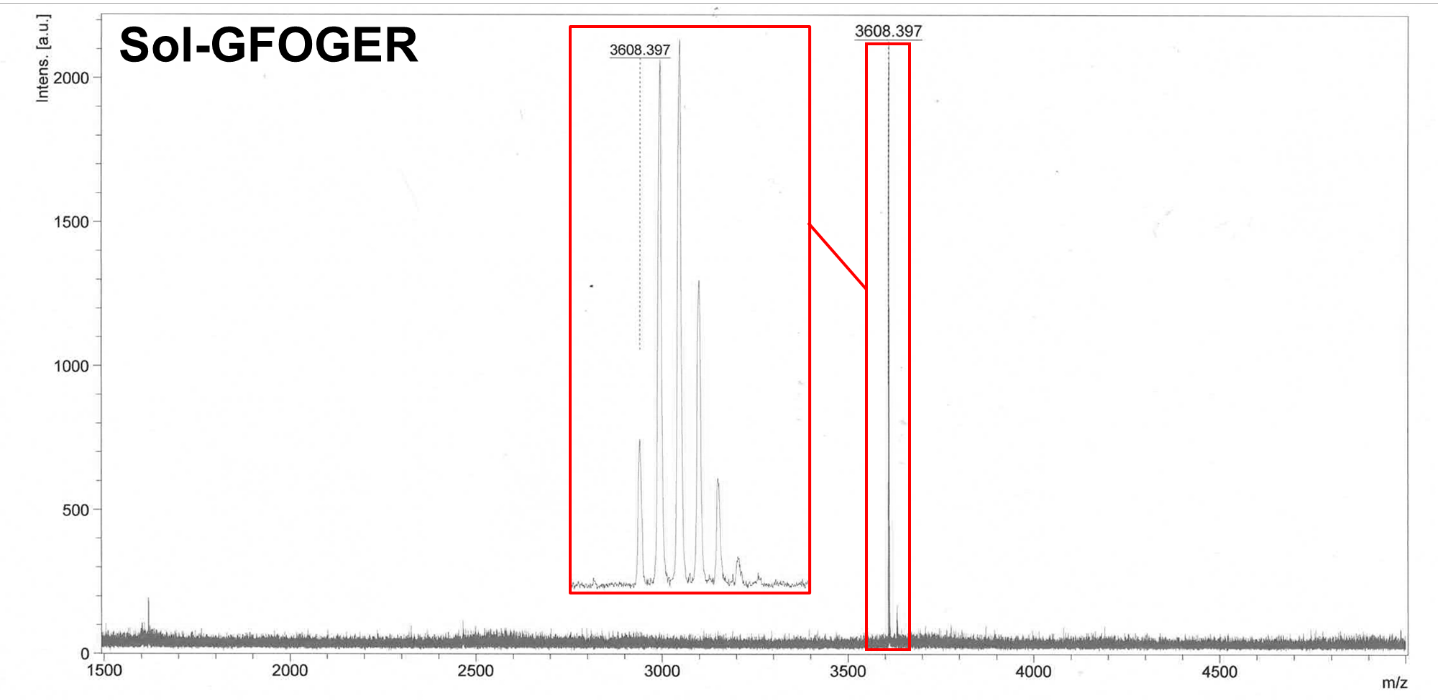


MALDI-MS m/z: [M+H]^+^ calculated for C_163_H_230_N_42_O_52_ = 3608.672, observed m/z = 3608.397

- 1. **CD profiles of the synthetic peptides**

**
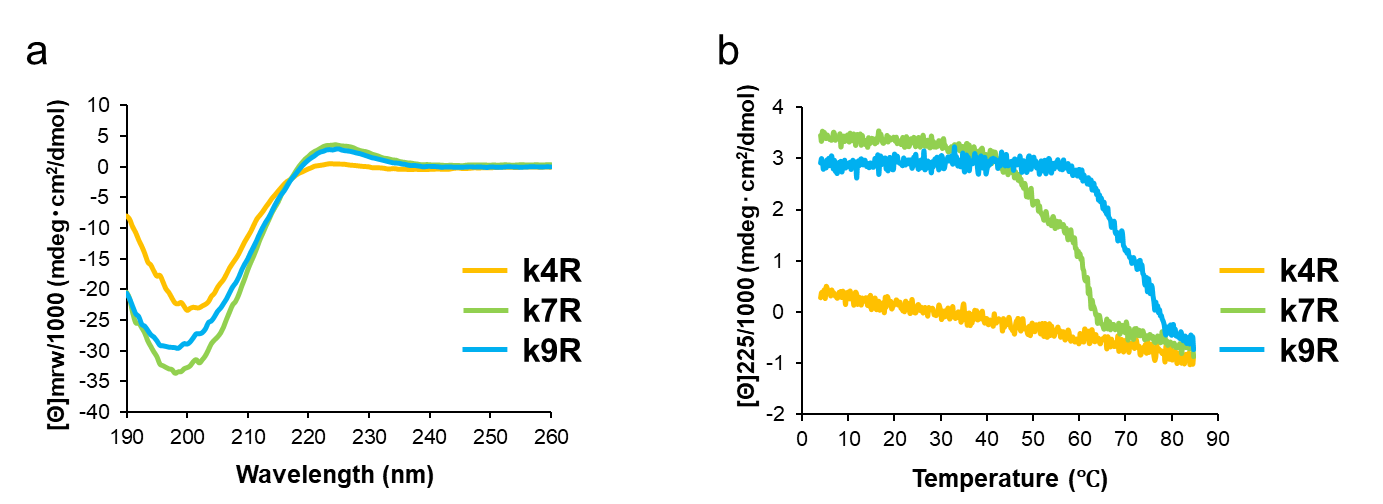
**

**Figure S3.** CD profiles of the synthetic peptides. (a) CD spectra recorded at 4 °C. (b) Thermal melting curves of the triple helices. The temperature was raised at a rate of 18 °C /h. Peptide solutions were prepared by dissolving each peptide in water at a concentration of 0.5 mg/mL, followed by annealing under the same conditions as those described in section 1.2.

- 1. **DLS analysis of the peptides**

**k4R k7R**


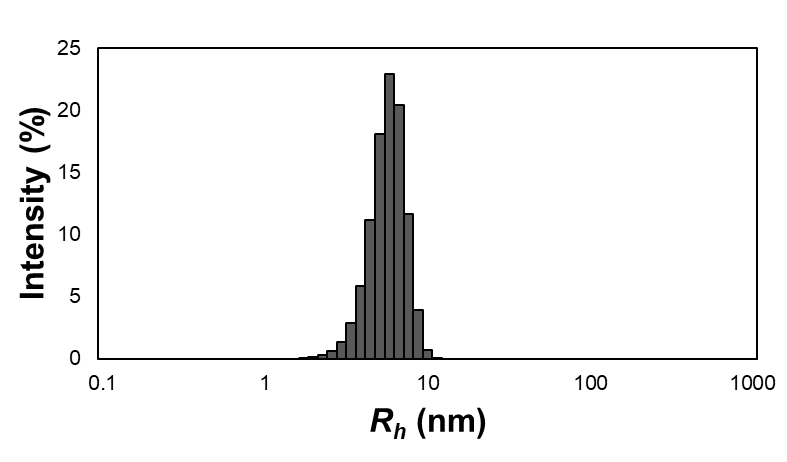

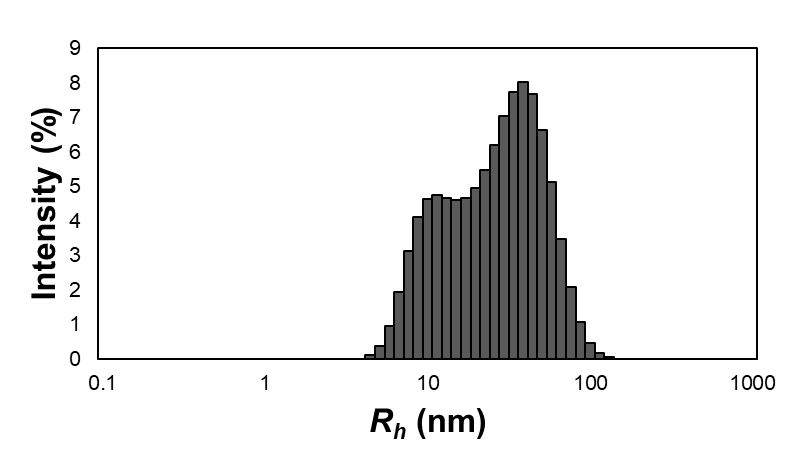


**k9R**

**
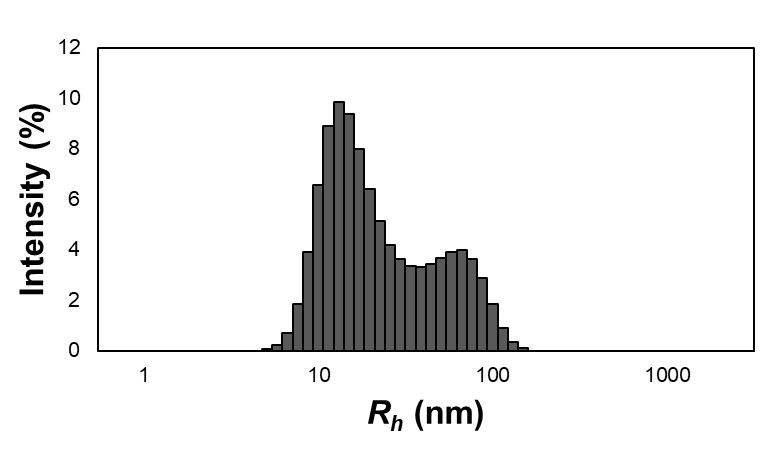
**

**Figure S4.** DLS analysis of the peptides

The particle size distributions were determined through DLS. Solutions of **k4R** and **k7R** (3.0 mg/mL each) and **k9R** (1.0 mg/mL) prepared in PBS were heated at 95 °C for 5 min, filtered through a 0.22 µm PVDF filter to remove insoluble materials, and stored at 4 °C overnight to allow them to fold.

- 1. **Thermal stability of GFOGER-containing k9R hydrogel**


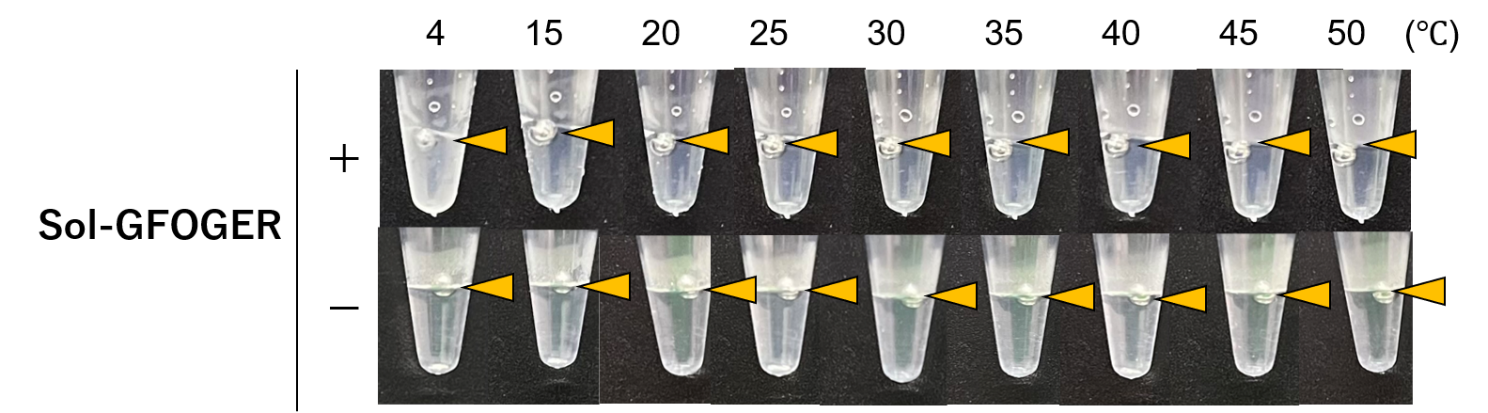


**Figure S5**. Thermal stability of GFOGER-containing **k9R** hydrogel

The thermal stability was evaluated for **k9R** hydrogels and mixed hydrogels of **k9R** and **Sol-GFOGER** prepared at a concentration of 20 mg/mL. The temperature was increased from 15 °C to 50 °C in 5 °C increments, with a 5 min equilibration at each temperature step.

- 1. **Stability of k9R hydrogel under the standard cell culture condition**


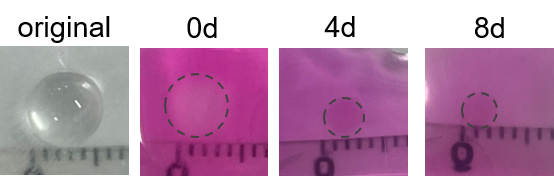


**Figure S6.** Time course of hydrogel size changes

A **k9R** hydrogel prepared at a concentration of 20 mg/mL was immersed in D-MEM supplemented with 10% (v/v) FBS, 100 units/mL penicillin, and 100 µg/mL streptomycin. The **k9R** hydrogel was incubated at 37 °C for 8 days in a humidified atmosphere. The dotted line outlines the gel within the culture medium. (1 division = 1 mm)

1. References

[1] J. Ottl, L. Moroder, *J. Pept. Sci.* **1999**, 5, 103-110.

[2] A. A. Adzhubei, M. J. Sternberg, A. A. Makarov, *J. Mol. Biol.* **2013**, *425*, 2100-2132.

[3] J. García de la Torre, J. G. Hernández-Cifre, *Eur. Biophys. J.* **2025**, *54*, 331-349.
